# Combined Liver and Inferior Vena Cava Resection for Malignancies Is Safe and Feasible in a Group of High-Risk Patients

**DOI:** 10.3390/jcm9041100

**Published:** 2020-04-12

**Authors:** Sandra Schipper, Markus Zimmermann, Andreas Kroh, Ulf Peter Neumann, Tom Florian Ulmer

**Affiliations:** 1 Department of General, Visceral and Transplantation Surgery, University Hospital of the RWTH Aachen, Pauwelsstr. 30, 52074 Aachen, Germany; akroh@ukaachen.de (A.K.); uneumann@ukaachen.de (U.P.N.); fulmer@ukaachen.de (T.F.U.); 2 Department of Nanomedicine and Theranostics, Institute for Experimental Molecular Imaging, RWTH University Clinic and Helmholtz Institute for Biomedical Engineering, 52074 Aachen, Germany; 3 Department of Diagnostic and Interventional Radiology, Aachen University Hospital, RWTH Aachen University, 52074 Aachen, Germany; mzimmermann@ukaachen.de; 4 Department of Hepatobiliary and Pancreatic Surgery, Maastricht University Medical Centre, 6229 HX Maastricht, The Netherlands

**Keywords:** vena cava, liver, malignancy, outcome

## Abstract

Background and Methods: Tumors infiltrating the inferior caval vein (ICV) have been considered irresectable in the past due to high perioperative risks. Consequently, the only treatment option for these patients was best supportive care, which resulted in reduced survival. Advancements in surgical techniques have since evolved, such that combined resections of the ICV and the hepatic malignancy are being performed. The aim of this study was the evaluation of the long-term outcomes (e.g., survival) and short-term risks of this procedure. In this single-center, retrospective cohort study (*n* = 24), we evaluated surgical and oncological outcome for patients undergoing hepatic surgery for oncological indications in combination with resections of the ICV. In addition, we investigated which factors are associated with survival. Results: First, we showed that perioperative mortality is as low as 4.1%. Second, we showed that perioperative co-morbidities are acceptable for this type of advanced hepatobiliary surgery. Third, the reconstruction of the ICV by means of a patch was superior in terms of survival compared to other types of reconstructions. This finding was independent of the type or the aggressiveness of tumor or the resections status. Discussion: In our cohort, many patients had undergone (multiple) preceding visceral surgical interventions or underwent multi-visceral surgery. Despite the medical complexity, survival was encouraging in this cohort, offering novel treatment modalities with a low risk of severe morbidities.

## 1. Key Points

— Combined hepatic resections and reconstruction of the ICV for oncological indications is a safe procedure.
— Combined reconstruction and hepatic surgery is a feasible and safe option even in extended surgery involving, e.g., the resection of the pancreas in the same surgical session.
— Reconstruction of the ICV by means of a patch is favorable in terms of survival independent of the completeness of the resection, the tumor differentiation, or the oncological indication.
— Perioperative mortality was as low as 4.1%. Most of the patients (33.3%) undergoing combined resections experienced no postoperative complications.
— The histopathological infiltration of the ICV is a better prognostic marker for survival than the completeness of the oncological resection or the differentiation of the primary tumor.
— Preoperative radiological assessment can aid in surgical and oncological decision making.

## 2. Introduction

Improvements in surgical techniques and management of hepatic malignancies have led to an increase in survival after surgery. A remaining challenge is the treatment of tumors infiltrating the inferior caval vein (ICV). In the past, patients with an infiltration of the ICV were considered poor candidates for surgery, their survival was limited, and the associated surgical risks were high [1]. Even stronger, infiltration of the ICV was a contraindication for surgical interventions. As such, the remaining interventions for this group of patients would consist of non-surgical techniques such as radio- or chemotherapy. However, these strategies are palliative and rarely curative [2]. The oncological resection of tumors is considered the gold standard for therapeutic intents. Generally, all tumors located in the upper right abdomen could infiltrate the ICV. Examples of these tumors are renal cell carcinomas [3], leiomyosarcoma, adrenal tumors, primary or secondary hepatic carcinomas (e.g., metastasis of gastrointestinal tumors), cholangiocarcinomas (CCC), hepatocellular carcinomas (HCC), and malignancies with a retroperitoneal manifestation, e.g., lymph node metastasis.

ICV resections require reconstructive procedures; their type depends on the extent of the resection. Reconstructive procedures involve primary reconstructions, e.g., by an end-to-end anastomosis, the use of synthetic or autologous patches, and reconstructions by means of an interposition graft made of, e.g., Polytetrafluoroethylene (PTFE) [4,5].

Surgically advanced techniques allowing the removal of the ICV improve resectability of advanced hepatic malignancies and therefore improve survival and reduce the probability of recurrence. The role different reconstructive procedures play in the long-term prognosis of patients with hepatobiliary surgery is unclear. Particularly, advances in surgical techniques enhance the complexity of patients who are candidates for surgery. Frequently, patients have undergone previous visceral interventions including surgical and radiological procedures.

Information about the risks and prognosis associated with resections of tumors infiltrating the ICV is therefore of crucial importance in informed decision making and patient counselling, including information about the perioperative risks, the chances of achieving a complete surgical resection, and the expected long-term outcome. Lastly, the rapid pace at which the hepatobiliary field is evolving has led to complex extended procedures, involving the resection of the liver, ICV, and other organs (e.g., pancreaticoduodenectomy).

We therefore investigated retrospectively all ICV resections that were performed in the course of liver surgery for oncological indications in our hospital with regard to multiple parameters such as perioperative morbidity and mortality, recurrence, and survival taking into account epidemiological and oncological characteristics. Furthermore, we specifically assessed the safety, long-term outcome, and feasibility in patients with extended and/or preceding surgeries. Moreover, we assessed whether preoperative radiological imaging can be used as a biomarker for ICV infiltration and predict individual risks and benefits.

## 3. Material and Methods

### 3.1. Subjects

We included patients who had undergone ICV resections in our hospital in the period from January 2010 to March 2018. Patients were selected based on the Diagnosis-Related group (DRG) code for ICV reconstructions. This means that only patients who received surgery were included. All surgeries took place at a tertiary academic center in Germany specialized in hepatobiliary surgery. In case operations were performed by another surgical discipline (e.g., urology) and not performed by an inter-disciplinary team in which a visceral surgeon participated, data were excluded. Consequentially, cases histories (discharge documentation, medical staff and nurses’ reporting, and radiological and pathological examinations) were reviewed by one person.

We included adult patients who were aged 18 years and above and excluded patients on whom surgery was performed less than six months ago, because of the short follow-up.

We retrieved the following parameters from the clinical database:

— Age and gender
— Oncological entity. To perform different subgroup analysis, we clustered patients based on their oncological indication into the following groups: sarcoma, cholangiocellular (including gallbladder cancer), or hepatocellular carcinoma (HCC; grouped as hepatobiliary cancer (HBC)), metastases of gastrointestinal tumors, or non-oncological indications.
— Type of liver surgery. We clustered patients into hemihepatectomies (left or right and extended), trisectionectomies, or segment resections.
— Type of vascular reconstruction of the ICV. Three types of reconstructions were considered, namely reconstruction by an interposition graft, patch reconstruction with a VASCU-GUARD bovine pericardium, or autologous patch, or primary closure (e.g., end-to-end anastomosis or tangential closure).
— Preceding visceral surgical interventions involving, e.g., the resection of a (primary) tumor.
— Extended simultaneous surgical procedures, which were performed in the same session as the combined resection. We classified them as “standard” surgical intervention or “extended” if any another type of visceral surgery took place.
— Liver specific laboratory parameters such as alanine aminotransferase (ALT), bilirubin, albumin, prothrombine time (PTT), and non-liver specific parameters such as creatinine and hemoglobin content were assessed at baseline.
— Tumor characteristics reported according to the Union for International Cancer Control (UICC) such as tumor grade of differentiation (G1, G2, and G3) and residual tumor (R) classification (R0: no residual tumor; R1: microscopic residual tumor).
— The absence or presence of histopathological invasion into the ICV upon postoperative evaluation by a trained pathologist.

We assessed all complications which occurred within 30 days after the operation (in hospital morbidity). We categorized these according to the internationally accepted Clavien–Dindo scale for the severity of complications. In all cases in which multiple complications occurred, the most severe category was leading for the analysis. Postoperative mortality was defined as death after surgery occurring during hospital stay.

### 3.2. Radiological Assessment of ICV Involvement

All preoperative Computed Tomographic (CT) images were assessed by a trained and experienced radiologist in a blinded manner. In conformity with the rating of portal vein invasion for pancreatic tumors, we used the criteria below to assess the degree of tumor invasion into the ICV [6].

— Tumor contact with the ICV, which could be categorized as a tumor with the convex contour pointing towards the vessel, a tumor with the concave contour pointing towards the ICV, or circumferential or irregular involvement.
— Length of the tumor contact with the vessel in cm.
— Degree of circumferential vein involvement.
— The degree of ICV compression in the categories: no stenosis, a flattened vein, or a stenosis by means of (near total) occlusion or radiological invasion into the ICV.

### 3.3. Follow-Up

All general practitioners were contacted by an independent, trained clinical investigator to retrieve patients’ survival status, date of death, and recurrence of the tumor. We also reviewed our clinical databases on whether patients had presented to the hospital by reviewing outpatient appointments, radiological examinations, and clinical discharge documents. For survival analysis, we excluded non-oncological cases, because inclusion would positively bias survival times.

### 3.4. Ethical Statement

All procedures were in accordance with the ethical standards of the responsible committee on human experimentation and with the Helsinki Declaration of 1975, as revised in 1983. The international and national guidelines regarding good clinical practice were followed.

### 3.5. Statistical Testing

All data were tested for normality and outliers, reaching a value higher or lower than two times the standard deviation of the mean, were excluded.

The association between categorical data was tested by a Pearson’s Chi-squared test with asymptotic significance correction. Continuous data were statically assessed with an independent student’s t-test or with a one-way ANOVA if there were more than two categorical variables involved. Survival times were assessed by means of Kaplan–Meier survival analysis. Differences between groups were tested by means of a log-rank test. For continuous covariates, survival analysis was performed by Cox-regression analysis with backward conditional modeling. A *p*-value of less than 0.05 was considered statistically significant. Statistical analyses were performed using SPSS statistical software (v.25; SPSS, Chicago, IL). Data are represented as means and standard error of the mean.

## 4. Results

**Figure 1:**
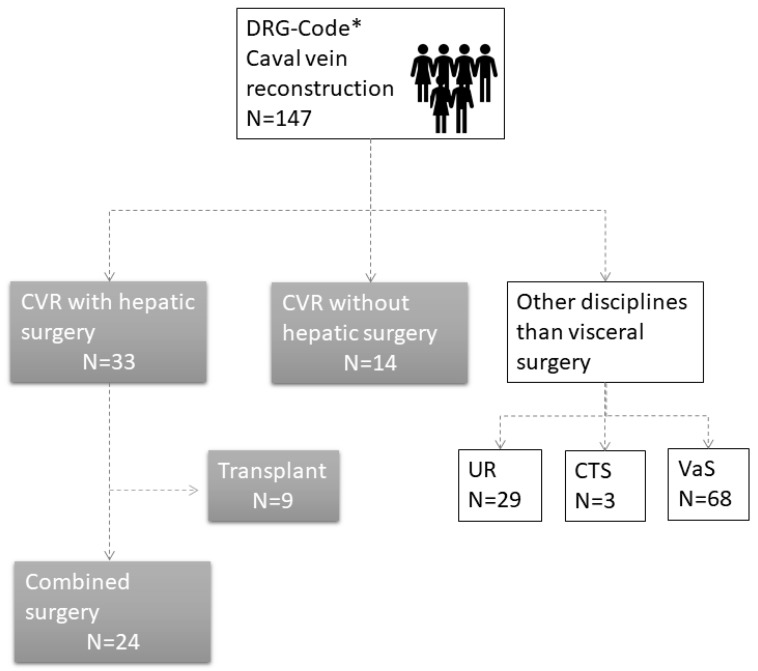
Flow diagram displaying the criteria for the inclusion in the study. In 147, patients the DRG code for ICV reconstruction was used. One hundred patients were operated by other disciplines and 14 received a reconstruction without hepatic surgery. Of the remaining 33 patients, 9 underwent liver transplantation. This resulted in a final sample size of 24 patients for combined surgical resections of the ICV and hepatic surgery. Abbreviations: CVR, caval vein reconstruction; UR, urology; VaS, vascular surgery; CTS, cardiothoracic surgery.

In total, 147 patients were included based on their DRG code. All patients (*n* = 100) who had surgery performed by other disciplines were excluded from the analysis. In total, 47 ICV reconstructions were performed. In 33 patients, the reconstruction was combined with hepatic surgery. Nine of these patients had liver transplantation. We excluded patients with a liver transplant because their prognostic parameters, perioperative morbidity, and epidemiological characteristics differ from oncological indications for ICV resections. Thus, 24 patients were included in this study (Figure 1). A detailed list with patients’ characteristics is displayed below.

### 4.1. Epidemiological Characteristics

Half of the patients were female (50%). The age at surgery ranged from 34 to 76 years with an average of 57.6 ± 2.4 years. Age at surgery was not significantly different between female and male patients (t(22) = 0.374; *p* = 0.712). The ASA classification was assessed by an experienced anesthesiologist and the presence of pulmonic, cardiac (expect for hypertonia), and nephrological co-morbidities is summarized in Table 1 below. Generally speaking, patients had a relative low ASA classification (II: *n* = 10, III: *n* = 14) and only three patients had cardiac or pulmonal diseases. The preoperative GFR was above 60 mL/min in all but four patients. No patients had severe kidney dysfunction.

Mean Body Mass Index (BMI) was 24.8 ± 0.99 kg/m^2^ (*n* = 24, range: 7.7–40.7 kg/m^2^). Only one patient was slightly underweight according to BMI measurements. Ten patients had a BMI above 25 and would therefore be classified as overweight. Eight patients received preoperative chemotherapy. The majority of patients receiving neoadjuvant therapy had hepatic metastasis (five out of nine).

### 4.2. Oncological Indication for Surgical Resection

The least frequent indications were for non-oncological causes (Echinococcosis, *n* = 1) and HCC (*n* = 2, 8.3%, of which one occurred in the presence of cirrhosis). The most frequent indication was CCC (*n* = 8, 33.3%) and (hepatic) metastasis of gastrointestinal tumors (*n* = 9, 37.5%). Four patients had a sarcoma (either leiomyosarcoma or sarcoma). Due to the small sample size, HCCs and CCCs were grouped together as hepatobiliary cancers (HBC, *n* = 10) for subgroup analysis. Indication for surgical resection and type of ICV repair did not vary over time, indicating no confounding by surgeons’ experience or preference.

### 4.3. Type of Hepatic Surgery

In 10 patients (41.7%), a right hemihepatectomy was performed. In three of these patients, the hemihepatectomy was extended and in one patient an additional segmental resection of Segment I was performed. In eight patients (33.3%), a segmental resection was performed. Three patients (12.5%) received a left hemihepatectomy (two of them extended) and three patients received a trisegmentectomy.

### 4.4. Extended Visceral Surgery

Fifty percent (*n* = 12) of the patients had undergone preceding visceral interventions. Six patients had hepatic surgery in the past. One patient received Step I of an Associating Liver Partition and Portal vein Ligation for Staged hepatectomy (ALPPS). One patient had a preceding tumor embolization and three patients received preoperative portal vein embolization (PVE).

Preceding surgery was not statistically significantly associated with postoperative complications, nor type of ICV reconstruction or completeness (R-status) of the resection.

In 41.7% of the patients, the resection of the ICV and the hepatic resection was combined with another procedure (e.g., pylorus preserving pancreatoduodectomies (PPPD), nephrectomies, tumorectomies, or additional vascular reconstructions).

### 4.5. Duration of Hospital Stay and Blood Transfusion

The average hospital stay was 30.3 ± 6.97 days; (Range: 7–160). Sixteen patients (66.7%) required intraoperative blood transfusion. The average number of intraoperatively applied number of packed red blood cells units was 6.33 (1.95, range 0–32). Eleven patients (45.8%) did not receive postoperative blood transfusions. The duration of hospital stay was significantly positively associated with the amount of intraoperative (r = 0.555, *p* = 0.005) and postoperative blood transfusions (r = 0.534, *p* = 0.007). In addition, the number of intraoperative blood transfusion was negatively associated with survival (Cox-regression: HR: 1.078, 95% CI: 1.014–1.146, *p* = 0.016).

### 4.6. Laboratory Parameters

All laboratory parameters which significantly changed (hemoglobin, albumin, ALT) at the third postoperative day, restored to normal at the fifth day. Only prothrombin time (PTT) was significantly decreased at the third (*p* ≤ 0.001) and fifth postoperative days (*p* = 0.0001; F(2, 17)= 32.208, *p* ≤ 0.001). Moreover, preoperative PTT (Cox-regression analysis HR: 0.840 95% CI: 0.743–0.950) was associated with survival and significantly correlated with the number of intraoperative blood transfusions (r = −0.463, *p* = 0.047).

### 4.7. Postoperative Complications

**Figure 2:**
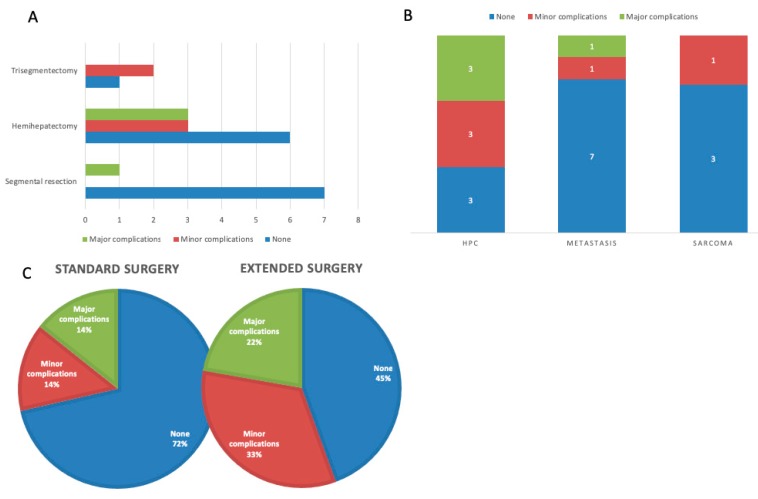
Occurrence of postoperative complications in relation to surgically relevant parameters such as extent of surgery, oncological indication and type of surgery performed. (**A**) Number of perioperative complications subdivided by type of hepatic resection. The number of patients having no complications is similar in all three types of hepatic surgeries performed. There was no statistically significant difference in the number of complications between different types of hepatic surgeries performed. (**B**) Number of perioperative complications subdivided by type of oncological indication. The patient with non-oncological indication did not experience complications. There was no statistically significant difference in the number of complications between different oncological indications. (**C**) Number of complications for different types of surgery either involving hepatic and caval surgery only (called standard surgery) or extended surgery involving multi-visceral procedures. The number of perioperative complications was not different between the two types of surgery performed.

One patient died on the fourth postoperative day due to a sepsis. The most frequently occurring postoperative complication was bile leakage, which was treated in most cases with a CT-guided drainage and/or percutaneous transhepatic biliary drainage (PTBD). One patient had a post-hepatic stenosis, which was treated with a PTBD. Major complications (bleeding and leakage of the biliodigestive anastomosis (BDA)) requiring re-surgery occurred in four patients (17.4%). Additionally, one patient showed a wound dehiscence requiring surgical intervention [7]. The number of patients experiencing postoperative complications according to Clavien-Dindo is displayed in Table 2. A graphical display of the complications by type of surgery and oncological indication is provided in Figure 2.

### 4.8. Recurrence

Information about recurrence within the follow-up period was available for 18 patients. In eight patients (44.4%), there was no recurrence. There was no statistically significant association of tumor recurrence with the following parameters: degree of differentiation (χ^2^ (2, *n* =15) = 1.111, *p* = 0.574), resection status of the primary tumor (χ^2^ (1, *n* =17) = 0.018, *p =* 0.893), histopathological infiltration into the ICV (χ^2^ (1, *n* =12) = 0.000, *p* = 1.000), and oncological indication for surgical resection (χ^2^ (2, *n* =18) = 3.463, *p* = 0.177). Histopathological evaluation confirmed invasion in 62.5%.

### 4.9. Survival Analysis

The follow-up time ranged from 7 to 93 months with a mean of 57.1 ± 5.34 months. Three patients with an oncological disease were lost to follow-up. At the time we performed the analysis, 13 patients were alive and 7 patients (excluding one postoperative death) had died. Of the seven patients who were alive, one had a distant metastasis and one patient recurrent leiomyosarcoma. Overall estimated median survival was 60.14 ± 19.50 months. The cumulative proportion of patients surviving at one, two, three, four, and five years was 95%, 79%, 61%, 61%, and 54%, respectively.

Preoperative ASA classification had no influence on survival (Log rank (Mantel–Cox): χ^2^ (2, *n* = 20) = 0.3392, *p* = 0.5603). In addition, there was no significant influence of preoperative chemotherapy on survival (Log rank (Mantel–Cox): χ^2^ (2, *n* =20) = 0.6014, *p* = 0.438). BMI was not significantly different between survivors and patients who died postoperatively (t(18)= 6.596, *p* < 0.751). There was also no linear correlation between survival time and BMI (r2 = 0.173, *p* < 0.419).

**Figure 3:**
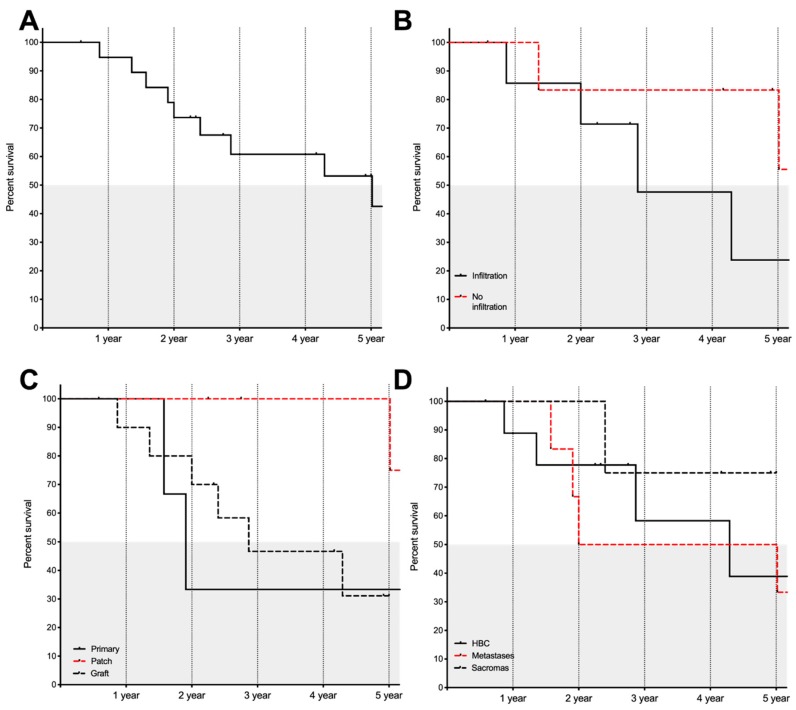
Kaplan–Meyer survival curves. On the x-axis the time in years is shown, while the y-axis shows the percentage of patients surviving. (**A**) Overall survival for all patients is shown. (**B**) The survival times in relation to the infiltrative status into the ICV are shown. There was a trend for improved survival in the group without infiltration into the ICV. (**C**) The survival status in relation to the type of reconstructive surgery of the ICV is shown. Patient with a patch reconstruction had a significantly better survival than patients receiving a graft or a primary closure of the ICV. (**D**) The survival in relation to the oncological indication is displayed.

Figure 3 shows overall survival, infiltrative status, type of reconstructive surgery being performed, and survival in relation to the oncological indication. In this dataset, survival was not influenced by the type of hepatic surgery performed (Log rank (Mantel–Cox): χ^2^ (3, n = 20) = 1.020, *p* = 0.797) or the completeness of the oncological resection (R0 vs. R1., (Log rank (Mantel–Cox): χ^2^ (1, n =19) = 0.341, *p* = 0.559) In addition, recurrence within the follow-up period did not significantly reduce survival times (Log rank (Mantel–Cox): χ^2^ (1, n = 16) = 0.424, *p* = 0.515). In addition, the extent of surgery in terms of volume had no influence on survival times (Log rank (Mantel–Cox): χ^2^ (2, n = 20) = 1–107, *p* = 0.575).

We assessed whether survival depends on the type of ICV reconstruction. Patient with a patch reconstruction had a significantly improved survival compared to other types of reconstructions (Log rank (Mantel–Cox): χ^2^ (2, n =20) = 6.108, *p* = 0.047). Patient with a patch reconstruction showed a median survival of 95.67 months whereas patients with a primary or graft reconstructions showed median survival of 22.91 and 34.41 months, respectively.

There was a statistically significant association between complete resections (R0) and the type of cancer (χ^2^ (2, n = 22) = 6.904, *p* = 0.032). All but one metastasis and all sarcomas were completely resected, whereas only 40% of the HBC were completely resected. Even though there was no improved survival in patients with a complete resection (Log rank (Mantel–Cox): χ^2^ (1, n = 19) = 0.341, *p* = 0.559), we hypothesized that it is easier to reach a complete resection with a graft, because a bigger portion of the ICV can be removed.

The occurrence of co-morbidities was no predictor of survival (Log rank (Mantel–Cox): χ2 (2, n = 19) = 3.915, *p* = 0.141).

### 4.10. Reconstruction of the ICV

Five (20.8%) patients received a primary reconstruction by a tangential closure or an end to end anastomosis. In seven patients (29.2%), a patch was used for the reconstruction. The majority of patients (n = 12, 50%) received an interposition xenograft. Four patients showed a thrombosis of the graft during radiological follow-up.

**Figure 4:**
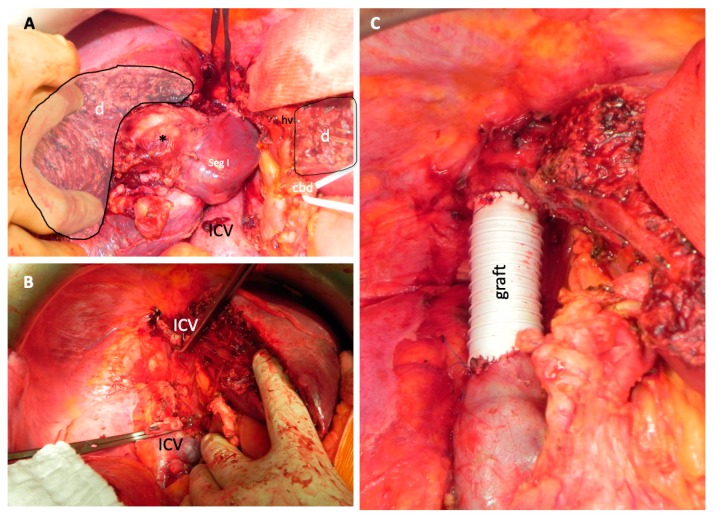
Intraoperative images obtained from a patient who received a GORE-TEX® interposition graft for the reconstruction of the ICV. The tumor, which was located in Segment I, infiltrated the ICV and the right pedicle. (**A**) The status after the dissection of the parenchyma (d) is shown. The middle hepatic vein (MHV) and the common bile duct (CBD) are located at the right side of the picture. The ICV is located at the bottom of the picture. (**B**) The status after the resection of the right liver with Segment I and the ICV, which is clamped in this picture, is shown. (**C**) The status after the reconstruction of the ICV with a GORE-TEX® graft is shown.

With all types of reconstructive procedures, a complete resection could be achieved (χ^2^ (2, n = 21) = 0.035, *p* = 0.983). HBC and sarcomas had a statistically higher chance of receiving a graft compared to patients with metastases (χ^2^ (4, n = 22) = 10.197, *p* = 0.037, see Table 3). Patients with sarcoma never received a patch reconstruction. In metastatic tumor manifestations most frequently, a primary reconstruction or patch reconstruction (n = 4 for both groups) was performed. Only one patient with a metastatic manifestation received a graft. However, in patients with CCCs and sarcomas, the most frequent type of reconstruction was a graft (six out of eight (75%) and four out of four, respectively). Intraoperative photographs of an interposition graft are shown in Figure 4. 

All ICV reconstructions had the same risk of postoperative complications (χ^2^ (4, n = 22) = 2.404, *p* = 0.662). 

None of the following parameters had an influence on the rate of complications: extent of surgery, gender, age at surgery, tumor type, type or volume of liver surgery, histopathological invasion into the ICV, resection status of the tumor, preceding surgical interventions, and type of vascular reconstruction.

### 4.11. Preoperative Radiological Assessment of Vena Cava Inferior Involvement

In one patient, no preoperative radiological assessment was performed, and the quality of the CT imaging was poor for one patient, leaving 22 patients for the analysis. All CTs were performed between 1 and 66 days prior to surgery (average: 26.22 ± 4.25 days).

None of the radiological parameters assessed predicted survival: tumor contact with the vessel (Log rank (Mantel–Cox): χ^2^ (2, n =19) = 2.866, *p* = 0.239); length of tumor contact (Cox-regression analysis: HR: 1.056, 95% CI: 0.847–1.315, *p* = 0.630); circumferential vein involvement (Log rank (Mantel–Cox): χ^2^ (1, n = 19) = 3.108, *p* = 0.375); and signs of stenosis (Log rank (Mantel–Cox): χ^2^ (2, n =19) = 1.859, *p* = 0.395). In addition, the length of tumor contact was not a predictor of survival (Cox-regression analysis: HR: 1.056, 95% CI: 0.847–1.315, *p* = 0.630).

The length of tumor contact was significantly influenced by the type of cancer investigated (F(2) = 5.993, *p* = 0.01). The length of HBCs and metastasis that was in contact with ICV was on average statistically significantly shorter (mean: 3.63 ± 0.6 cm, *p* < 0.029 and 3.06 ± 0.63 cm, *p* = 0.01, respectively) compared to sarcomas (7.75 ± 2.02 cm).

However, type of contact and the presence of ICV stenosis were a good predictor for the type of reconstructive surgery (tumor contact: χ^2^ (4, n = 21) = 11.926, *p* ≤ 0.018; ICV stenosis: χ^2^ (4, n = 21) = 10.575, *p* = 0.032). In the case of no stenosis or a tumor pointing with the convex contour towards the vessel, 60% of the reconstructions were performed with a patch. In the case of vein occlusion (five out of eight) or circumferential involvement > 270 degrees (five out of ten), most reconstructions were performed by means of an interposition graft. None of the radiological parameters could predict infiltration into the ICV.

## 5. Discussion

The proportion of patients with HBC undergoing combined resections surviving at five years in our dataset is 42%. For comparison, patients who did not receive adjuvant treatment with advanced hepatocellular carcinoma had a median survival of 7.9 months [8]. In addition, very aggressive chemotherapy regimens with a severe impact on the quality of life showed a median survival of 22.1 months in advanced biliary tract cancer [9]. Considering the fact that patients who are considered irresectable have no alternative treatment modalities, the here presented outcome is clearly superior compared to palliative care. Previously, it had been described that invasion of HCC into the ICV decreases survival 1–4 months [10]. About 4% of the patients with, e.g., hepatocellular carcinomas, show infiltration into the ICV [11]. The worldwide incidence of HCC in 2012 was 14 million and is estimated to rise to 22 million in the next decade [12]. This means that 560,000 patients were candidates for ICV resections for HCC only. Due to the anatomical vicinity of the tumor to the ICV, the number of patients benefiting from combined resections in CCC is probably much higher.

In the current study, only a few cases with HCC were included. For the present analysis, we grouped HCCs and CCCs as primary hepatic malignancies into the group of hepatobiliary cancers. Even though both have a poor prognosis, their estimated survival differs and therefore grouping them might bias survival times.

Owing to the limited number of medical centers performing combined resections and the feared high risk of this interventions, most studies only include small numbers of patients and conclusions about the superiority of a combined surgical approach for long-term survival and morbidity are ethically challenging. In a meta-analysis by Zhou et al. [13], 258 patients were included. They evaluated the safety and efficacy of combined liver and ICV resections for oncological indications. They concluded that combined resections are safe and effective. Most of the patients who were included in this meta-analysis were patients without preceding surgical interventions or surgeries. To our knowledge, the current study is the largest study in this specific subset of patients, who are at high risk of perioperative morbidity and mortality due to their medical complexity.

Zhou et al. described postoperative morbidity to be 43% and the necessity for re-laparotomy in 2% of the included patients. In our dataset, 12 patients (50%) experienced severe morbidities categorized as Clavien–Dindo grade III or higher. One third of the patients did not experience any postoperative complication. Strikingly, Zhou described liver failure in 35% of the patients. In our dataset, none of the patients experienced liver failure.

Five-year survival for patients with curative resections for HCC that is not invading into ICV has been reported to be up to 57% [14]. In our dataset, five-year overall survival for patients with HBC was 54%. These results are especially promising when considering this particular patient group, which was in the past considered irresectable and would therefore most likely receive best supportive care or palliative chemotherapy.

Furthermore, Zhou reported median survival for combined resections to be 34 months. The one-, three-, and five-year survival rates were 79%, 45%, and 31%, respectively. For comparison, median survival in our cohort was nearly double (60 months) and one-, three-, and five-year survival rates were 95%, 61%, and 54%, respectively. Moreover, the follow-up in our study was much longer (median 60 months compared to 22 months). Considering that our patient group had frequently undergone multiple (surgical) interventions before the combined surgery and that we performed multi-visceral surgical procedure in a subset, these survival times are encouraging. One explanation could be that our dataset included patients with HBC, metastases, and sarcomas. Sarcomas are generally more favorable in terms of survival compared to cancers of hepatic origin or metastases of gastrointestinal tumors, which might in part explain the good results in this study. However, the oncological indication for surgical resection was not significantly associated with survival.

None of the included studies in the meta-analysis of Zhou reported preoperative morbidity (i.e., reported by the American Society of Anesthesiologists (ASA) class), thus we were unable to compare this factor. However, it is possible that the selection of patients for this type of advanced surgery is not free from bias. Patients with a low ASA category are likely more prone to undergo more complex procedure such as combined resections. In the present study, patients were included retrospectively after surgery had been performed. We did not evaluate patients who had ICV invasion and were not operated. This might explain why in our cohort patients were relatively healthy (ASA II and III), which might in turn explain the promising survival data and the low number of postoperative complications. The fact that we analyzed patients retrospectively who underwent surgery might per se create a selection bias, because potentially healthier patients are more likely to undergo extended surgeries. With the rapid advances in perioperative and surgical techniques, it is likely that reduced surgery time, less invasive procedures, etc. will allow surgery to be performed on multi-morbid patients, too. The current results are encouraging the application of combined resections in patients with a higher ASA classification.

A drawback of this study is the small sample size, which causes a lack of statistical power for some sub-group analysis. Even though this study is one of the bigger series in the field and the first to focus on patients that underwent multiple surgical procedures simultaneously and a proportion even had preceding surgical interventions, we acknowledge that patient characteristics were heterogeneous.

However, we found that patients with negative tumor margins are performing equally well in terms of survival compared to patients with positive margins. This finding seems at first contradictory, but more studies found that neither overall survival nor recurrence rates do correlate with R-Status [15,16]. This finding might be related to the fact that patients with positive tumor margins are more likely to receive postoperative. Due to the organization of the health care system in Germany, chemotherapy and oncological follow-up are frequently executed by practitioners, which made it difficult to retrieve reliable data whether the advice of the interdisciplinary tumor board was obeyed. However, we provide information about the advice for additive therapies on an individual patient basis as a surrogate marker for potential follow-up treatment. 

We also found that there was a significant association between the tumor entity and the resection status. HBC had a lower chance of being completely resected compared to metastases or sarcomas. Considering that survival for HBC is generally worse than that of sarcomas, this finding might constitute a confounder itself. However, in our dataset survival did not differ significantly between tumor entities. Currently there is no consensus whether resection status is a predictor of overall survival (see Torzilli et. al., 2019 or Donadon et al., 2019) [17,18].

In addition, the invasion into the ICV, which intuitively would be a marker of more invasive cancers, only correlated weakly with survival. These findings are in line with the meta-analysis previously discussed, where the authors reported a trend in respect to survival for gender and type of hepatectomy (major vs. minor). We could duplicate the finding that females show improved survival rates but found no effect of the resected volume on survival.

Strikingly, we found a strong effect of the type of vascular reconstruction. Patch reconstructions were superior in terms of survival. More surprisingly, this benefit was independent from most of the commonly used prognostic factors with regard to survival such as recurrence, differentiation of the tumor, oncological indication, or completeness of the resection.

We hypothesized that graft reconstructions were a more aggressive type of surgery involving a bigger resection area resulting in more complications, and in turn limited short-term survival. However, the rate of complications was similar for all reconstructive procedures. Moreover, the occurrence of complications per se was not a prognostic factor.

One risk of interposition graft is that these can become non-patent by a thrombosis. In our clinic, patients receive therapeutic antithrombotic treatment immediately after surgery until four weeks postoperative. All other patients receive prophylactic low dose heparin for the same period. The added value of routinely using therapeutic anticoagulative therapy is controversial and still under debate. In addition, it is unclear, if the occurrence of graft thrombosis influences survival. In other studies, it has been shown that graft patency in hepatic malignancies is 100% in the first 22 months [13]. In addition, in our dataset, the number of non-patent grafts in the follow-up period was low. The long-term patency of grafts in hepatic malignancies is unknown and therefore it would be speculative to assume that graft patency played a role in these patients after 22 months.

Another mechanism possibly explaining a difference in survival between different types of reconstructions is the fact that graft placement requires clamping of the ICV causing liver ischemia. However, survival curves for grafts and primary reconstructions, which mostly do not require (long) clamping, are very similar, suggesting that ischemic time is not a predictor of survival either. In the future, it would be of added value to investigate what factors play a role in the improved long-term survival in patients with patches. In the present study, patients only received synthetic grafts. Because of their improved safety and improved long-term patency without the necessity for anticoagulation, the use of biological grafts (e.g., peritoneo-fascial or bovine pericardial graft) has been proposed [19]. The investigation of vascular dynamics, time of clamping, biomaterials, and alterations in liver perfusion and their effect on survival is an interesting topic for future research.

Moreover, we were interested whether preoperative radiological imaging can be used for clinical decision making. Unfortunately, most parameters failed to predict survival. However, the type of tumor contact with the vessel and the presence of a stenosis were predictive for the type of reconstruction performed, which in turn correlated with survival.

In the current study, we assessed which factors serve as biomarkers for survival in patients with a complex medical background and found that female gender, no infiltration into the ICV (statistical trend), and the reconstruction of the ICV by means of a patch were positively associated with survival. These factors can be used in preoperative decision making and patient counseling. Moreover, we propose to use postoperative histological evaluations about the infiltration status for the individual and oncological risk assessment of patients in the decision making about adjuvant chemotherapy.

Comparisons about the superiority of combined surgical approaches to a liver only approach in terms of survival are difficult to make, because it is unethical to refrain from ICV resection in the presence of macroscopic ICV invasion. Therefore, the question whether survival is improved in hepatic resection only will be unanswered. However, perioperative mortality, which in the past has frequently been considered as a reason not to perform surgery, was only 4.1%, which is an acceptable mortality for this type of advanced liver surgery. Moreover, the risk found in our study is comparable to the risk that has been reported by others [13].

In summary, oncological radical resections can be considered the optimal and only treatment strategy with therapeutic intention. The oncological resection of tumors infiltrating the ICV has been a challenge for many years, because of the associated perioperative risks and mortality. We could show that extended surgery with vascular reconstructions can be performed safely and efficiently in challenging surgical cases. Combined resections should be offered in hepatobiliary centers to a heterogeneous group of patients taking into account individual factors in the preoperative risk assessment.

## Figures and Tables

**Table 1:** Individual characteristics of the included patients. Epidemiological data as well as data about the type of procedure performed and the postoperative course are provided.

	Gender	Age	Type of Tumor	Previous Visceral Interventions	Simultaneous Procedures	Complications	Preoperative Chemotherapy	Additive Chemotherapy	ASA	Comorbidity	Resection Status	BMI	Survival Status (Months)	Type of Surgery
1	M	37	Metastasis	—	Hemicolectomy (right), Ureter resection, partial peritonectomy	—	None	Yes	II	None	R1	23.4	Dead (23)	Segmentresection
2	M	64	Sarcoma	Tumor embolization	PPPD, Nephrectomy, Pankreatikogastrostomy, Gastrojejunostomy	Spontaneous bacterial peritonitis	None	No	III	None	R0	22.1	LTF	Hemihepatectomy right
3	M	67	Metastasis	PVE Hemicolectomy left and LAR Segment resection IV	—	—	Yes	-	II	None	R0	23.5	Alive (19)	Trisegmentectomy
4	F	53	Metastasis	PPPD	—	Bile leak→Drainage	None	-	II	None	R0	25.7	Alive (96)	Hemihepatectomy right
5	M	57	CCC	PVE Resection of the sigmoid colon	—	Bile leak →Drainage	Yes	Yes	III	None	R0	21.3	Alive (16)	Trisegmentectomy
6	M	69	CCC	Hemihepatectomy, partial adrenalectomy, diaphragmatic resection, reconstruction of hepatic artery and portal vein	Reconstruction of hepatic artery, renal and portal vein, diaphragmatic resection, Hepaticojejunostomy Roux-Y-Anastomosis, Nephrectomy	BDA leak → **Re-surgery**	Yes	-	III	C	R0	23.9	Alive (34)	Segmentresection
7	M	62	Metastasis	Resection of the sigmoid colon, Atypical Seg VI resection PVE	Reconstruction hepatic fork	—	Yes	Yes	II	None	R1	27.4	LTF	Hemihepatectomy right
8	F	73	Metastasis	Resection of the sigmoid colon		Cholecystitis	Yes	No	III	Hypertonia		26.3	Alive (24)	Segmentresection
9	F	34	Metastasis	LAR Extended hemihepatectomy right, Segment III resection	Diaphragm resection	Seropneumothorax	Yes	No	III	None	R0	22.3	Alive (147)	Segmentresection
10	F	65	CCC	*In situ* split, Portal vein reconstruction BDA with Roux-Y-Anastomosis	—	Fascial dehiscence	None	Yes	III	None	R0	28.0	Alive (11)	Hemihepatectomy right extended
11	M	48	Metastasis	—	PPPD, Reconstruction of portal vein	Leak of the pankreaticojejunostomy anastomosis Fistula, Laparostoma **Re-surgery** Colon perforation and bleeding CT drainage	None	No	II	None	R1	27.8	Alive (60)	Hemihepatectomy right
12	F	76	Sarcoma	Multiple tumor resections, Patch reconstruction of caval vein, nephrectomy	PPPD	—	None	No	III	None	R0	23.5	Dead (59)	Segmentresection
13	M	62	Metastasis	Atypical segmental resection V & VIII, LAR	—	—	Yes	No	III	None	R1	28.4	LTF	Hemihepatectomy left extended
14	F	62	Sarcoma	—	—	—	Yes	No	III	None	R0	40.7	Dead (60)	Segmentresection
15	F	60	CCC	—	BDA	**Bleeding→Re-surgery** Bile leak →Drainage	None	Yes	III	None	R0	21.9	Alive (51.5)	Hemihepatectomy left
16	F	65	Gallbladder	Atypical hepatic resection Pulmonal wedge – resection	PPPD, BDA Pankreatiokojejunostomy, Gastrojejunostomy Reconstruction of renal vein	Bile leak→Drainage	None	No	III	None	R0	23.3	Alive (121)	Trisegmentectomy
17	F	47	Sarcoma	Tumor resection Caval vein resection	Nephrectomy, Adrenalectomy		None	No	II	None	R0	19.4	Dead (50)	Segmentresection
18	M	57	HCC	—	Reconstruction of portal vein, BDA	PTBD Wound healing disorder	None	Yes	II	None	R0	24.2	Alive (79)	Hemihepatectomy left extended
19	F	42	CCC	—	Diaphragm resection	—	None	No	III	None	R1	17.7	Dead (33)	Hemihepatectomy right
20	F	56	CCC	—	Bile duct reconstruction and Resection of Seg. I	—	None	Yes	III	None	R1	26.9	Dead (28)	Hemihepatectomy right
21	F	47	CCC	—	Reconstruction of portal vein, Diaphragmatic resection, BDA	Bile leakage, Bleeding, **Re-surgery**, Sepsis, PTBD Death	None	-	III	None	R1	19.3	Alive (4.3)	Hemihepatectomy right
22	M	40	Non-oncological	—	—	—	None	-	II	None	R0	32.1	LTF	Hemihepatectomy right extended
23	M	66	HCC	Stenting of ductus choledochus	—	—	None	No	III	C+P	R1	19.2	Dead (27)	Hemihepatectomy right extended
24	M	73	Metastasis	—	—	—	None	No	II	P	R0	27.1	Dead (7)	Segmentresection

Used Abbreviations: LAR: Lower anterior resection, PVE: Portal vein embolization, BDA: Biliodigestive anastomosis, PPPD: Pylorus Preserving Pancreatoduodectomy, PTBD: percutaneous transhepatic biliary drainage, LTF: lost to follow-up, P: pulmonal comorbidity, c: cardiac comorbidity..

**Table 2:** Number of patients experiencing postoperative complications according to Clavien–Dindo grades.

Clavien–Dindo Grade	Description	Number of Patients
**None**		9
**I**	Any deviation from expected clinical course	1
**II**	Requiring pharmacological treatment	3
**III**	Requiring interventions	9
**IIIa**	Without anesthesia	5
**IIIb**	With anesthesia	4
**IV**	Life-threatening complications	1
**V**	Death	1

**Table 3:** The number of patients receiving a specific type of ICV reconstruction by oncological indication. In the HBC and the sarcoma groups, the reconstruction by means of a graft prevails, whereas patients with metastases were less likely to receive a graft.

Type of Reconstructive Procedure	Metastasis	Sarcoma	HBC	Total
**Primary reconstruction**	4	0	1	5
**Patch reconstruction**	4	0	3	7
**Graft**	1	4	6	11
